# Effect of Model of Neonatal Care on Neurodevelopment at the 18 Month Follow-Up in Moderate and Late Preterm Infants

**DOI:** 10.3390/jcm14020586

**Published:** 2025-01-17

**Authors:** Karen M. Benzies, Fiona C. Bartram, Deborah A. McNeil

**Affiliations:** 1 Faculty of Nursing and Departments of Pediatrics and Community Health Sciences, Cumming School of Medicine, University of Calgary, 2500 University Drive NW, Calgary, AB T2N 1N4, Canada; 2 Faculty of Nursing, University of Calgary, 2500 University Drive NW, Calgary, AB T2N 1N4, Canada; fiona.bartram@ucalgary.ca; 3 Faculty of Nursing and Department of Community Health Sciences, Cumming School of Medicine, Alberta Health Services, University of Calgary, 2500 University Drive NW, Calgary, AB T2N 1N4, Canada; debbie.mcneil@albertahealthservices.ca

**Keywords:** moderate and late preterm infant, family integrated care, randomized controlled trial, child global development, child socio-emotional development, maternal psychosocial distress, follow-up study

## Abstract

**Background:** Preterm birth, even for moderate or late preterm infants (MLPIs), is associated with longer-term developmental challenges. Family Integrated Care (FICare) models of care, like Alberta FICare, aim to improve outcomes by integrating parents into neonatal care during hospitalization. This follow-up study examined the association between models of care (Alberta FICare versus standard care) and risk of child developmental delay at 18 months corrected age (CA) and explored the influences of maternal psychosocial distress. **Methods:** We assessed 257 mothers and 298 infants from a cluster randomized controlled trial (ID: NCT0279799) conducted in ten Level II NICUs in Alberta, Canada. Risk of delay was assessed using developmental screening tests. Maternal psychosocial distress was assessed using self-reported measures of depressive symptoms, anxiety, parenting stress, and self-efficacy. **Results:** There was no association between model of care and risk of developmental delay. Higher maternal parenting stress was associated with increased risk of developmental delay. **Conclusions:** Alberta FICare was not associated with decreased risk of developmental delay at 18 months CA. Maternal parenting stress may play an important role in the development of MLPIs and should be addressed post-discharge.

## 1. Introduction

Globally, preterm birth affects approximately 10% of live births [1]. Approximately 15% of preterm infants are born very preterm, at <32 weeks gestational age (GA); the remaining 85% are moderate or late preterm infants (MLPIs), born between 32 weeks and zero days and 36 weeks and 6 days GA [1,2]. Due to advances in neonatal intensive care, preterm infants are more likely to survive; however, many have morbidities [3]. As gestational age (GA) decreases, the risk of morbidity increases [4,5]. Compared to their full-term counterparts, MLPIs are at higher risk for (1) poor health (e.g., respiratory, growth, and feeding problems) [6,7,8], (2) poor developmental outcomes (e.g., neurodevelopmental disabilities, cognitive and language delays, and school-related problems) [6,9,10], and (3) emotional and behavioral problems [9,11,12,13]. At school entry, 8.3% of MLPIs have developmental delays, including fine motor, communication, and personal-social skills [14]. These deficits likely stem from early disruptions in neural development and postnatal environmental influences, which creates greater risk for developmental delay in MLPIs.

At birth, MLPIs often require care in a neonatal intensive care unit (NICU). NICUs are critical care environments where healthcare providers often unintentionally marginalize parents in the pursuit of lifesaving care of the infant [15,16]. The unexpected birth of a preterm infant often leaves parents in shock, feeling anxious, depressed, isolated, and unprepared to interact with, and care for, their newborn [15,17]. Preterm birth and experiences in the NICU also disrupt human milk feeding [18] and early parent–infant relationships [19,20], which are necessary conditions for optimal child development [20,21,22]. While maternal mental health does not affect their involvement in the care of very preterm infants while in hospital [23], the negative effects of the postnatal family environment on preterm child development after discharge is clear. For example, in a Danish population cohort population cohort (N = 19,017), postpartum depression, as measured by the Edinburgh Postnatal Depression Scale, and preterm birth (30 to 36 weeks’ gestation) predicted infant social withdrawal up to an infant age of 12 months [24]. Social withdrawal is considered prodromal to socio-emotional problems in childhood and mental illnesses in adulthood. In a narrative review, low maternal education and social disadvantage also negatively influenced cognitive, motor, and behavior development in children born at <28 weeks’ gestation [25]. Thus, preterm birth and family environment are associated with a complex variety of negative outcomes for the infant.

Family-integrated models of care aim to educate and support parents to become members of the NICU team [26]. Healthcare provider and parent roles shift dynamically as parents become more knowledgeable and confident in caring for their infant [27]. However, interventions to improve developmental outcomes in preterm infants delivered in the NICU and/or post discharge have demonstrated mixed results [28,29]. Several studies have demonstrated benefits to child development of integrating families into their preterm (<33 weeks’ gestation) infant’s care team during their Level III NICU stay [30,31,32]. In a systematic review and meta-analysis, the authors found that preterm (<37 weeks) infants who received a variety of interventions (e.g., environmental stress controls, individualized developmental care, and maternal education to understand infant behavioral cues) had significantly better cognitive and motor outcomes at 12 months and better psychomotor development at age 24 months [33]. This meta-analysis was limited by the heterogeneity of interventions and few randomized controlled trials. In another systematic review and meta-analysis of RCTs of family-centered care interventions in the NICU, the authors found no effect on infant neurobehavioral development, although this review included only three RCTs that assessed neurobehavioral development [34]. However, the included studies dated back to 2006 and pre-dated two cluster RCTs with positive results of developmental follow-up [35,36]. In a follow-up study of a Canadian subsample (N = 126) from a 25-site cluster randomized controlled trial (FICare n = 895; standard care n = 891) with preterm infants <33 weeks’ gestation [30], the authors found group differences in child internalizing and externalizing behavior problems favoring FICare, which were mediated by maternal hair cortisol levels but not subjective measures of stress [37]. Taken together, these results suggest that interventions to support preterm infant development that are delivered in the NICU may be effective for early preterm infants, but positive effects may erode over time. Also, most previous studies were conducted in Level III NICUs; none were focused exclusively on MLPIs, who typically receive care in a Level II NICU. Thus, follow-up studies on the association of models of neonatal intensive care and risk of developmental delays in MLPIs are needed to optimize models of care of these infants and their families in the NICU.

### 1.1. Objectives

The objective of this study was to examine the association of models of neonatal intensive care (Alberta FICare versus standard care) and the risk of child development in MLPIs at age 18 months corrected age (CA). The primary research question was as follows: Compared to standard care in a Level II neonatal intensive care unit (NICU) and controlling for covariates, does Alberta FICare influence child global development of MLPIs at 18 months as measured by the Ages and Stages Questionnaires, 3rd Edition (ASQ-3) [38]. The secondary research questions were as follows: Compared to standard care in a Level II NICU and controlling for covariates, does Alberta FICare influence child social emotional and behavioral development at 18 months CA as measured by the Ages and Stages Questionnaires: Social-Emotional, 2nd Edition (ASQ:SE-2) [39], and the Brief Infant Toddler Social Emotional Assessment (BITSEA) [40]. As covariates, we used child and maternal characteristics that have been identified as contributing to child development. Maternal characteristics included depressive symptoms, anxiety, parenting stress, and self-efficacy, as measured by the Center for Epidemiologic Studies Depression Scale—Revised (CESD-R), [41] the State Trait Anxiety Inventory (STAI) [42] State Anxiety subscale, the Parenting Stress Index, 4th Edition Short Form (PSI-4-SF) [43], and the General Self-Efficacy (GSE) [44] scale. We hypothesized that, after controlling for child and maternal characteristics, Alberta FICare would decrease the risk of developmental delay at age 18 months CA.

### 1.2. Theoretical Framework

We applied Bronfenbrenner’s [45] theory of bio ecological development to this study. Bronfenbrenner asserts that multi-factorial interactions between individuals and micro-, meso-, exo-, and macro-systems influence children’s development. Specifically, at the micro-system level, interactions occur between individuals internal or external to the family. At the meso-system level, interactions occur between two or more micro-systems, such as family and health care settings. At the exo-system level, interactions between events and processes, and family and healthcare settings are included. Finally, at the macro-system level, culture is embedded across each of the previous systems and influences child development. Thus, child development is complex and influenced by multiple factors at multiple levels in their environment.

## 2. Materials and Methods

### 2.1. Study Design and Setting

This study was the 18-month-old follow-up to our cluster randomized controlled trial (cRCT) evaluating the effects of Alberta FICare in ten Level II NICUs in Alberta, Canada [27]. See clinicaltrials.gov ID: NCT0279799. Entire NICUs were randomized to five Alberta FICare sites and five standard care sites. At the time of this study, Alberta had a single integrated healthcare system serving a population of 4.4 million people, with approximately 50,000 births per year [46]. Alberta Health Services espoused the philosophy of family centered care [47]: none of the sites had previously implemented a family integrated model of care.

### 2.2. Sample

**Figure 1:**
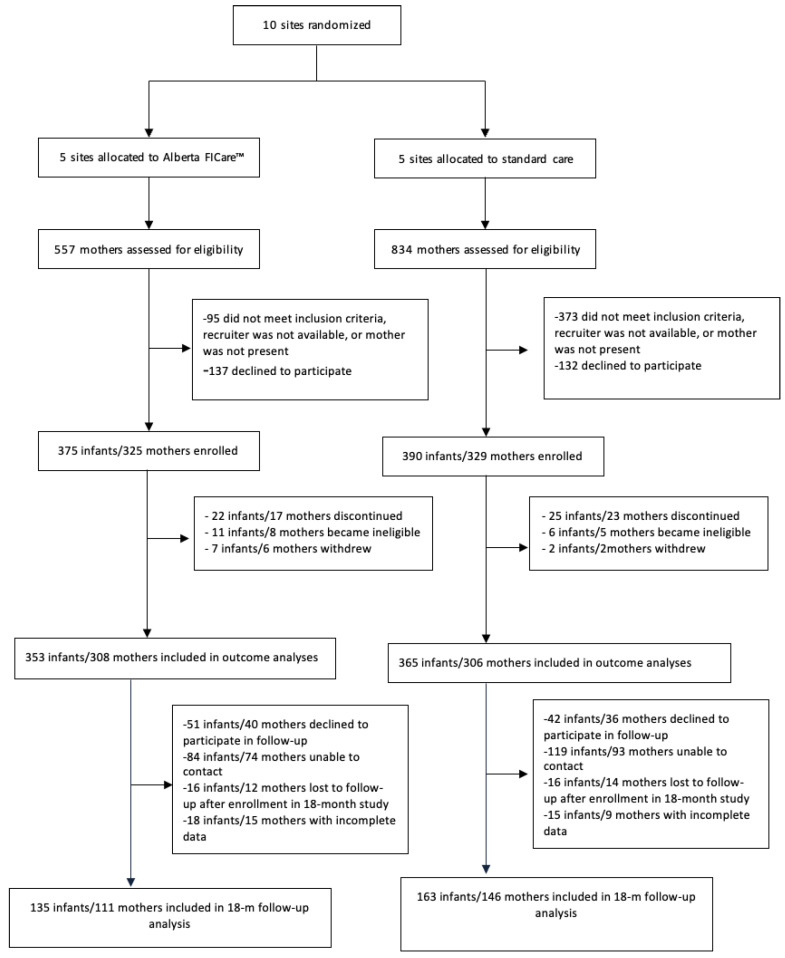
CONSORT flow diagram. Note: FICare = Family Integrated Care.

For the cRCT, we recruited mothers and their singleton or twin preterm infants born between 32 weeks and 0 days and 34 weeks and 6 days GA between December 2016 and July 2018 [27]. GA was based on the 12-week ultrasound. The upper limit of GA was selected to ensure that infants received at least one week exposure to Alberta FICare, because otherwise healthy MLPIs who are gaining weight are often discharged from Canadian NICUs at 36 weeks and zero days. Discharge criteria may vary by country. We excluded mothers with language or social issues as well as infants who required palliative care or had severe congenital or chromosomal anomalies. Follow-up data were collected between October 2017 and March 2020. Of the 614 mothers of 718 infants included in the cRCT outcome analyses, there were 257 mothers of 298 children included in the 18-month follow-up study. See Figure 1, CONSORT flow diagram. The final sample consisted of 111 mothers of 135 children in the Alberta FICare group and 146 mothers of 163 children in the standard care group. Compared to mothers who did not participate in the 18-month follow-up study, mothers who participated were more likely to be partnered (96.9% versus 91.7%, *p* = 0.01), Caucasian (76.0% versus 61.9%, *p* < 0.001), have post-secondary education (83.1% versus 74.8%, *p* = 0.02), and report an annual family income over $80,000 CAD (66.9% versus 47.1%, *p* < 0.001).

### 2.3. Intervention

**Figure 2:**
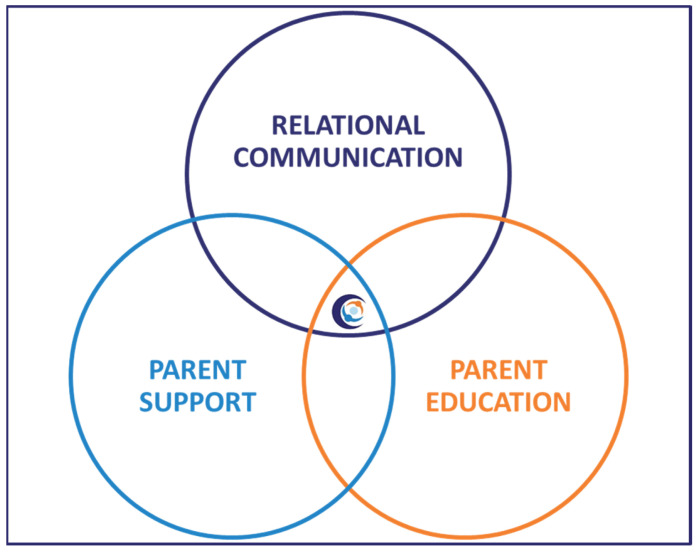
Components of Alberta Family Integrated Care.

Alberta FICare is a psycho-educational intervention with three components, which empower parents to sequentially build their knowledge, skill, and confidence so they are well prepared to care for their preterm infant before discharge. See Figure 2. Parents are educated and coached to provide routine, non-medical care; healthcare providers continue to provide medical and technical care, such as intravenous medications and procedures, legal documentation, and professional support for families [48]. In the cRCT, compared to infants in the standard care group, infants in the Alberta FICare group were discharged 2.55 days sooner without concomitant increases in emergency department visits and readmissions [27]. When Alberta FICare was scaled to all 14 NICUs in the province, there were lower-than-expected lengths of stay, emergency department visits, and readmissions [49]. 

### 2.4. Measures

See Table 1 for characteristics of measures used in the follow-up study. Maternal psychological distress was measured at 18 months using the Center for Epidemiological Studies Depression scale—Revised (CESD-R) [41], State Trait Anxiety Scale (STAI) [42], and Parenting Stress Index (PSI) [43]. Child age was adjusted for prematurity for all measures of child development.

### 2.5. Procedures

To retain participants in the study, we maintained contact through quarterly newsletters to participants and a birthday card sent to the individual child. Approximately one month prior to the child turning 18 months CA, research assistants contacted eligible mothers to inquire about their interest in the follow-up study. For interested mothers, we sent a link to an electronic informed consent form. After mothers completed the consent form, we sent a link to the surveys, which were completed at admission and discharge and at 18 months CA. For mothers who did not complete the survey within two weeks, we sent two reminder emails. At 18 months, eleven mothers completed the online survey outside the time frame for the ASQ-3. In those cases, we excluded the ASQ-3 scores from analysis; however, we retained scores on the other measures, as they were valid. Six children were already past 18 months CA when recruitment for this follow-up study started. Therefore, four mothers completed the 20-month version, and two mothers completed the 22-month version of the ASQ-3

### 2.6. Data Analysis

We examined data for missing values and patterns of missing values. Where possible, we replaced missing values according to scale developers’ recommendations. In the absence of recommendations, we replaced up to 20% of the missing values with the group mean on that item. We analyzed data using Chi-square tests for categorical variables and *t*-tests for continuous variables. We estimated Pearson’s bivariate correlations between child development risk on three assessments (ASQ-3, ASQ:SE-2, and BITSEA) and potential covariates (infant and maternal characteristics). Independent variables with correlations of *p* < 0.01 were included in the final logistic regression models. The *p*-value was set to 0.05. IBM SPSS Version 28 was used for statistical analyses.

## 3. Results

### 3.1. Child and Maternal Characteristics by Group

In the follow-up sample, there were significant differences between groups in the proportion of singletons and maternal ethnicity. For the follow-up sample, mothers in the Alberta FICare group reported significantly more singleton births and Caucasian ethnicity than mothers in the standard care group. There were no other group differences in child or maternal socio-demographic characteristics. See Table 2.

### 3.2. Risk of Child Developmental Delay and Maternal Scale Scores

See Table 3 for frequencies and percentages of risk for developmental delay on the ASQ-3, ASQ:SE-2, and BITSEA domains by group (Alberta FICare versus standard care). See Table 4 for scores on maternal depressive symptoms, anxiety, parenting stress, and self-efficacy scales. In three logistic regression models, we examined the associations between maternal socio-demographic characteristics, maternal psychosocial distress, maternal self-efficacy, and group with child global development (ASQ-3) and social and emotional difficulties (ASQ:SE-2 and BITSEA). We found that mothers who were not partnered reported higher risk of global delay (ASQ-3) and social-emotional problems (ASQ: SE-2) for their child. Mothers who were born in Canada reported a lower risk of social-emotional difficulties (ASQ:SE-2 and BITSEA) and behavioral problems (BITSEA). Mothers who reported higher parenting stress reported a higher risk of global delay (ASQ-3) and of social emotional difficulties (ASQ:SE-2 and BITSEA) and behavioral problems (BITSEA). Maternal age, education, depressive symptoms, anxiety, and self-efficacy did not contribute to the risk of child developmental delay at 18 months CA. Contrary to our hypothesis, group did not contribute significantly to risk of child developmental delay. See Table 5.

See Supplementary Table S1 for Pearson’s correlations to identify covariates that were included in the models. Except for group, which we hypothesized would make a difference in risk of child developmental delay at age 18 months, we included covariates in the models that were significant at *p* < 0.01. No other variables that were collected at admission, discharge, or 18 months were associated with risk of child developmental delay.

## 4. Discussion

In this follow-up study of mothers and their MLPIs from a cRCT (N = 654), Alberta FICare in Level II NICUs was not associated with decreased risk of child global development delay or social-emotional and behavioral difficulties at age 18 months CA. However, increased maternal parenting stress was associated with increased risk of developmental delay on each measure. The results of our follow-up study of MLPIs in Level II NICUs contrast with results from two follow-up studies of early preterm infants (<33 weeks’ gestation) from a cRCT (N = 1786) of FICare in Level III NICUs [37,50]. In a subsample (N = 123) between 18 and 21 months, FICare was associated with lower child dysregulation, suggesting that FICare had a sustained positive effect on infant behavior [50]. In a similar subsample (N = 126) at 18 months, FICare was associated with decreased child internalizing and externalizing problems, and this association was mediated by lower maternal stress hormones [37]. One explanation for the lack of significant differences in the risk of developmental delay between MLPIs in Level II NICUs and early preterm in Level III NICUs may be related to the relatively shorter length of stay of MLPIs. A shorter length of stay limits exposure to FICare practices and may reduce the potential for a longer-term influence of the model of care. Also, MLPIs generally have fewer health complications than early preterm infants, reducing the opportunity for interventions like FICare to influence their neurodevelopment [6]. 

### 4.1. Maternal Psychosocial Distress and Child Development

In contrast to the results of a systematic review and meta-analysis of psycho-educational interventions for parents of preterm infants that identified positive effects on maternal depression and stress [51], in our study, Alberta FICare was unrelated to maternal psychosocial distress. Shorter length of NICU stay, and the limited exposure of mothers of MLPIs to the model of care may explain why there was no association between Alberta FICare and maternal psychosocial distress.

In our follow-up study, maternal parenting stress was associated with increased risk of child developmental delay for both the Alberta FICare and standard care groups. These results suggest that maternal psychosocial distress after discharge may have a stronger influence on the developmental outcomes of MLPI infants than exposure to FICare. These results are consistent with other studies that suggest post-discharge environments and maternal factors play a more significant role in a child’s development than interventions in the NICU [52,53].

### 4.2. Maternal Self-Efficacy and Child Development

In our study, Alberta FICare was not related to maternal general self-efficacy. In contrast, a systematic review reported that psychosocial interventions in the NICU and community increased self-efficacy for mothers of preterm infants, but only up to 6 months [28]. It is possible that any positive influence of Alberta FICare on maternal self-efficacy eroded by 18 months. Alternatively, in our study we used a measure of general self-efficacy. The use of a measure specific to parenting self-efficacy for a preterm infant may have yielded different results.

### 4.3. Maternal Demographic Characteristics and Child Development

In our follow-up study, maternal marital status and whether the mother was born in Canada were associated with the risk of child developmental delay. Partnered mothers reported fewer developmental concerns for their child compared to mothers who were parenting alone. Our results are consistent with previous research with mothers of infants (N = 2023) with a birth weight of 1250 g [54]. Compared to mothers in two-parent families, mothers who parented alone were younger and had less education and income [54]. At age 3 years CA, infants living in lone-parent families had greater diagnosed mild/moderate impairments [54]. In a systematic review, the quality of family (defined as > two individuals) interactions was more important for cognitive and behavioral development in preterm than term children [55]. This review did not include studies of families with lone-parent mothers, who face greater emotional and financial stress. Together, these results reinforce the need for supportive interventions for interactions in lone-parent families to improve developmental outcomes, even for children who are born moderate and late preterm. In our study, mothers who were not born in Canada reported higher levels of child social-emotional and behavioral difficulties. This may be influenced by differences in values, beliefs, and parenting practices in different cultures [56]. Divergence in developmental expectations that parents have for their children in their current context and country of origin may conflict. These differences may explain why mothers who were not born in Canada reported higher levels of social-emotional and behavioral difficulties for their child.

### 4.4. Limitations and Future Research

Strengths of this follow-up study include a larger sample than many previous studies, which is likely to decrease selection bias. However, loss to follow-up resulted in low completion rates and significant group differences in singleton versus twin births and ethnicity at 18 months. Loss to follow-up also resulted in a smaller-than-expected sample size. With small group differences (15.1% versus 12.5%) on the primary outcome (proportion at risk for delay on the ASQ-3), this study was underpowered to find group differences. In addition, this study included rigorous measurement of maternal characteristics and child development using reliable and valid scales. However, this study has several limitations. While FICare encourages parental involvement during the NICU stay, it may not adequately address the post-discharge needs of families. Future research should explore extending parental support after discharge, especially in addressing maternal psychosocial distress and parenting self-efficacy. While we measured whether mothers were partnered, we did not measure the quality of support from the partner. Future studies should assess the quality of social support from the mother’s partner. Additionally, this study relied on self-reported data for maternal psychosocial distress, which could introduce bias. Objective measures, such as physiological indicators of stress (e.g., cortisol levels), may provide more accurate assessments in future studies [37]. Moreover, this study focused exclusively on MLPIs, who generally have better developmental outcomes compared to early preterm infants [14]. Future studies should explore whether different subgroups of preterm infants benefit more from FICare interventions, particularly those born at earlier gestational ages, who may experience more severe developmental challenges. Assessment at 6, 12, and 18 months CA would provide information about erosion of the influence of Alberta FICare on child and maternal outcomes. A longer follow-up period, beyond 18 months, may also provide insights into the enduring effects of FICare on developmental outcomes at school age and beyond [14]. 

## 5. Conclusions

In conclusion, while the Alberta FICare model provides valuable support to mothers of MLPIs during the NICU stay, post-discharge maternal psychosocial distress and family environments played a more important role in determining risk of global and social-emotional developmental delays. Interventions targeting maternal psychosocial distress and parenting self-efficacy, particularly after discharge, may be key to reducing the risk of child developmental delays. Future research should aim to further understand the complex interplay between NICU interventions and post-discharge family environments, with a focus on developing longer-term support systems for mothers of MLPI infants with psychosocial distress.

## Figures and Tables

**Table 1:** Characteristics of measures.

Measure	Description
Ages and Stages Questionnaires, 3rd Edition (ASQ- 3) [38]	Widely used developmental screening instrument consisting of 21 age-appropriate questionnaires for use with infants and children 2 to 60 months of age. Each questionnaire has 30 items that assess risk of developmental delay across five domains: communication, gross motor, fine motor, problem solving, and personal-social. For each domain, age-specific cut-off scores have been empirically derived for appropriate development, monitoring zone (1 and > standard deviations below the mean), or referral required (1 standard deviation below the mean). Sensitivity (0.86) and specificity (0.85) are high. For this study we use referral in any domain to denote risk.
Ages and Stages Questionnaires: Social-Emotional, 2nd Edition (ASQ:SE-2) [39]	Widely used screening instrument for social and emotional difficulties in infants and children 2 to 60 months of age. Consists of 9 age-appropriate questionnaires, with about 30 items per questionnaire. Total scores are compared to empirically derived age-specific cut-offs for appropriate social-emotional development, monitoring zone (≥1 and <2 standard deviations below the mean), or referral required (≥2 standard deviations below the mean). Sensitivity (0.81), specificity (0.84), test–retest reliability (0.89), and internal consistency reliability (0.84) are all high. For this study, we collapsed monitoring and referral categories to denote risk.
Brief Infant Toddler Social Emotional Assessment (BITSEA) [40]	Valid and reliable screening instrument designed to identify emerging social-emotional and behavioral problems in children between the ages of 12 and 36 months. Consists of 42 items and provides total scores on two subscales: Problem Behavior and Competence. Age and gender-specific cut-off scores have been empirically derived for each subscale to identify children at risk of delay in social-emotional development. For this study, we collapsed risk on problem and competence subscales to denote risk.
Center for Epidemiologic Studies Depression Scale—Revised (CESD-R) [41]	Self-report measure of depressive symptoms for the general population. Consists of 20 items rated on a 4-point Likert scale. Items are summed to calculate a total score; higher scores indicate greater depressive symptoms. Theoretical scores range from 0 to 60. Internal consistency reliabilities (0.85 to 0.90) are high, and test–retest reliabilities (0.45 to 0.70) are moderate.
State-Trait Anxiety Inventory (STAI) [42]	Self-report measure of current (state) and dispositional (trait) anxiety. Consists of 40 items, 20 per subscale, rated on a 4-point Likert scale. Items are summed to calculate a total score for each subscale; higher scores indicate greater anxiety. Internal consistency (0.86 to 0.95) and test–retest reliabilities (0.73 to 0.86) are high. Only state anxiety was measured in this study.
Parenting Stress Index, 4th Edition Short Form (PSI-4-SF) [43]	Self-report measure of parenting stress across three domains: parental distress, parent–child dysfunctional interaction, and difficult child. Consists of 36 items rated on a 5-point Likert scale. Items are summed to calculate domain scores and a Total Stress score. Theoretical scores range from 12 to 60 for each domain and 36 to 180 for Total Stress; higher scores indicate greater parenting stress. Internal consistency reliability coefficients (0.95 for Total Stress and 0.88 to 0.90 for subscales) are high. Test–retest studies were not conducted for this version of the instrument.
General Self- Efficacy (GSE) [44]	Self-report measure of self-efficacy consisting of 10 items measured on a 4-point Likert scale. Theoretical scores range between 10 and 40. Items are summed to calculate a total score; higher scores indicate greater self-efficacy. Internal consistency reliability is high (0.76 to 0.90 across samples).

**Table 2:** Comparison of child and maternal characteristics by Alberta FICare versus standard care group.

		Alberta FICare		Standard Care	P(χ^2^)
	No.	Frequency (%)	No.	Frequency (%)	
**Child Characteristics**					
Singleton (% yes)	163	129 (79.1)	135	87 (64.4)	0.005
Gestational age at birth (% yes)	163		135		0.463
32 weeks		41 (25.2)		29 (21.5)	
33 weeks		48 (29.5)		35 (25.9)	
34 weeks		74 (45.4)		71 (52.6)	
Male (% yes)	163	86 (52.8)	135	77 (57.0)	0.460
Caesarean delivery (% yes)	163	76 (46.6)	135	69 (51.1)	0.441
IV fluids (% yes)	163	89 (54.9)	131	54 (41.2)	0.025
**Maternal Characteristics**					
Relationship status (% yes)	147		116		0.597
Single		7 (4.8)		4 (3.4)	
Partnered		140 (95.2)		112 (96.6)	
Employment (% yes)	148		116		0.231
Employed full or part time		108 (73.0)		86 (74.1)	
Homemaker/not in the labor force		26 (17.6)		24 (20.7)	
Unemployed and looking for work		8 (5.4)		1 (0.9)	
Other ^a^		6. (4.1)		5 (4.3)	
Education (% yes)	160		134		0.846
High school diploma or lower		28 (17.5)		22 (16.4)	
Postsecondary certificate/diploma		43 (26.9)		33 (24.6)	
College/university degree		89 (55.6)		79 (59.0)	
Annual family Income (CAD) (% yes)	159		132		0.782
<$40,000		7 (4.4)		8 (6.1)	
$40,000 to $79,999		22 (13.8)		20 (15.2)	
≥$80,000		108 (67.9)		90 (68.2)	
Prefer not to answer/don’t know		22 (13.8)		14 (10.6)	
Born in Canada (% yes)	160	131 (81.9)	134	103 (76.9)	0.289
Ethnicity (% Caucasian)	161	132 (81.0)	134	93 (69.4)	0.011

Note: Sample size varies due to missing data. ^a^ Other includes maternity leave, student, and on disability.

**Table 3:** Frequencies and percentages of risk for child developmental delay by group.

Child Assessments	No.	Alberta FICare	No.	Standard Care
		Frequency (%)		Frequency (%)
**ASQ-3 Domains**				
Communication	154		129	
Referral		4 (2.6)		1 (0.8)
Gross Motor	154		129	
Referral		11 (7.1)		5 (3.9)
Fine Motor	154		129	
Referral		8 (5.2)		3 (2.3)
Problem Solving	152		127	
Referral		14 (9.2)		6 (4.7)
Personal-Social	154		129	
Referral		3 (1.9)		2 (1.6)
**ASQ-3 Any Domain**	152		128	
Referral		23 (15.1)		16 (12.5)
**ASQ:SE-2**	157		122	
Referral		10 (6.4)		7 (5.7)
**BITSEA**				
Problem Behavior	155		124	
Risk		27 (17.4)		22 (17.7)
Competence	155		124	
Risk		11 (7.1)		15 (12.1)
Any Subscale	155		124	
Risk		33 (21.3)		30 (24.2)

Note: Sample size varies due to missing data. Abbreviations: ASQ-3, Ages and Stages Questionnaires, 3rd Edition; ASQ:SE-2, Ages and Stages Questionnaires: Social-Emotional, 2nd Edition; BITSEA, Brief Infant Toddler Social Emotional Assessment. aASQ-3 and ASQ:SE-2 risk collapsed monitoring and referral categories into one risk category.

**Table 4:** Means and standard deviations on maternal scales by group.

	No.	FICare Mean (SD)	No.	Standard Care Mean (SD)
**Maternal scales**				
CESD-R	151	7.40 (8.62)	118	6.47 (8.86)
STAI State Anxiety	151	32.70 (10.18)	117	32.86 (10.84)
PSI-4-SF				
Total Stress	152	61.53 (17.13)	113	64.89 (19.85)
Parental Distress subscale	152	23.74 (8.02)	113	24.84 (9.32)
Parent-Child Dysfunctional Interaction subscale	152	17.52 (5.55)	113	18.72 (5.78)
Difficult Child subscale	152	20.28 (5.95)	113	21.34 (6.93)
GSE	151	33.06 (4.53)	117	33.23 (4.39)

Note: Sample size varies due to missing data. Abbreviations: CESD-R, Center for Epidemiologic Studies Depression Scale, Revised; STAI, State-Trait Anxiety Inventory; PSI-4-SF, Parenting Stress Index, 4th Edition Short Form; GSE, General Self-Efficacy.

**Table 5:** Logistic regression models for associations between maternal socio-demographic characteristics, maternal psychosocial distress, maternal self-efficacy, and group and child global development (ASQ-3) and social emotional and behavioral difficulties (ASQ:SE-2 and BITSEA).

Variable	ASQ-3	ASQ:SE-2	BITSEA
	aOR (95% CI)	aOR (95% CI)	aOR (95% CI)
Constant	0.110	17.053	12.578
Socio-demographics			
Marital status	0.255 (0.64, 1.009) *	0.118 (0.021, 0.662) **	0.896 (0.211, 3.800)
Maternal age	1.043 (0.968, 1.124)	0.958 (0.847, 1.083)	0.953 (0.893, 1.018)
Maternal Education	0.895 (0.522, 1.535)	0.641 (0.275, 1.496)	0.379 (0.474, 1.151)
Born in Canada	0.836 (0.328, 2.130)	0.192 (0.055, 0.675) **	0.373 (0.174, 0.802) **
Maternal psychosocial distress			
CESD-R	0.981 (0.928, 1.037)	0.993 (0.928, 1.061)	1.012 (0.961, 1.066)
STAI	1.000 (0.949, 1.054)	0.977 (0.908, 1.052)	0.976 (0.931, 1.023)
PSI (total)	1.046 (1.017, 1.075) **	1.061 (1.017, 1.107) **	1.033 (1.009, 1.057) **
Maternal self-efficacy			
GSE	0.993 (0.893, 1.103)	0.979 (0.837, 1.144)	0.937 (0.863, 1.017)
Group	0.692 (0.309, 1.548)	0.601 (0.166, 2.175)	1.049 (0.542, 2.029)

Note: Abbreviations: aOR: adjusted odds ratio; CI: confidence interval. ASQ-3, Ages and Stages Questionnaires, 3rd Edition; ASQ:SE-2, Ages and Stages Questionnaires: Social-Emotional, 2nd Edition; BITSEA, Brief Infant Toddler Social Emotional. * *p* < 0.05; ** *p* < 0.01.

## Data Availability

Data are available to qualified researchers upon reasonable request to the corresponding author.

## Supplementary Materials

The following supporting information can be downloaded at: https://www.mdpi.com/article/10.3390/jcm14020586/s1, Table S1: Pearson’s correlations for modeled variables.

## Abbreviations

ASQ-3	Ages and Stages Questionnaire version
ASQ:SE-2	Ages and Stages Questionnaire version
BITSEA	Brief Infant and Toddler Social Emotional Assessment
CA	corrected age
CESD-R	Center for Epidemiological Studies Depression scale, Revised
FICare	Family Integrated Care
GA	gestational age
GSE	General Self-Efficacy Scale
MLPI	moderate and late preterm infant
NICU	neonatal intensive care unit
PSI-4-SF	Parenting Stress Index version—short form
STAI	State Trait Anxiety Scale
