# Supplementary material for: Effect of Model of Neonatal Care on Neurodevelopment at the 18 Month Follow-Up in Moderate and Late Preterm Infants
Source: J Clin Med. 2025 Jan 17;14(2):586. doi: 10.3390/jcm14020586 (PMC11766077; doi:10.3390/jcm14020586)
Supplement: Supplementary file 1 [file jcm-14-00586-s001.zip › jcm-3374721-supplementary.pdf]

Supplementary Table S1. *Pearson's correlations of modelled variables.*

	2	3	4	5	6	7	8	9	10	11	12	13
**1. ASQ**	.268***	.312***	-.110	-.139*	.033	-.16	-.068	.121	.175**	.268***	-.132*	-.038
**2. ASQSE**	--	.441***	-.095	-.161**	-.137*	-.147**	-.123*	.209***	.253***	.373***	-.205***	.013
**3. BITSEA**		--	-.126*	-.022	-.094	-.098	-.165**	.179**	.172**	.299***	-.211***	.035
**4. IV Fluids**			--	-.022	-.160**	-.014	.039	-.003	-.056	-.70	.030	-.136*
**5. Marital Status**				--	.056	.118	.038	.007	-.057	-.027	-.015	.033
**6. Maternal Age**					--	.302***	-.107	-.042	.031	-.032	.012	.080
**7. Maternal education**						--	-.099	-.011	-.085	.006	.048	.029
**8. Born in Canada**							--	.019	.005	-.093	.037	-.062

**9. CESD-R**	--	.697***	.623***	-.380***	-.053
**10. STAI**		--	.654***	-.467***	.008
**11. PSI**			--	-.438***	.091
**12. GSE**				--	.019
**13. Group**					--

Note: Abbreviations: ASQ-3, Ages and Stages Questionnaires, 3rd Edition; ASQ:SE-2, Ages and Stages Questionnaires: Social-Emotional, 2nd Edition; BITSEA, Brief Infant Toddler Social Emotional Assessment; CESD-R, Center for Epidemiologic Studies Depression Scale Revised; STAI, State-Trait Anxiety Inventory; PSI-4-SF, Parenting Stress Index, 4th Edition Short Form; GSE, General Self-Efficacy.
*p < .05; **p < .01; ***p < .001
